# Cross-Modal Functional Reorganization of Visual and Auditory Cortex in Adult Cochlear Implant Users Identified with fNIRS

**DOI:** 10.1155/2016/4382656

**Published:** 2015-12-27

**Authors:** Ling-Chia Chen, Pascale Sandmann, Jeremy D. Thorne, Martin G. Bleichner, Stefan Debener

**Affiliations:** ^1^Neuropsychology Lab, Department of Psychology, European Medical School, Carl-von-Ossietzky University Oldenburg, 26129 Oldenburg, Germany; ^2^Department of Neurology, Hannover Medical School, 30625 Hannover, Germany; ^3^Cluster of Excellence Hearing4all, 26129 Oldenburg, Germany; ^4^Research Center Neurosensory Science, University of Oldenburg, 26129 Oldenburg, Germany

## Abstract

Cochlear implant (CI) users show higher auditory-evoked activations in visual cortex and higher visual-evoked activation in auditory cortex compared to normal hearing (NH) controls, reflecting functional reorganization of both visual and auditory modalities. Visual-evoked activation in auditory cortex is a maladaptive functional reorganization whereas auditory-evoked activation in visual cortex is beneficial for speech recognition in CI users. We investigated their joint influence on CI users' speech recognition, by testing 20 postlingually deafened CI users and 20 NH controls with functional near-infrared spectroscopy (fNIRS). Optodes were placed over occipital and temporal areas to measure visual and auditory responses when presenting visual checkerboard and auditory word stimuli. Higher cross-modal activations were confirmed in both auditory and visual cortex for CI users compared to NH controls, demonstrating that functional reorganization of both auditory and visual cortex can be identified with fNIRS. Additionally, the combined reorganization of auditory and visual cortex was found to be associated with speech recognition performance. Speech performance was good as long as the beneficial auditory-evoked activation in visual cortex was higher than the visual-evoked activation in the auditory cortex. These results indicate the importance of considering cross-modal activations in both visual and auditory cortex for potential clinical outcome estimation.

## 1. Introduction

Modern cochlear implants (CI) allow deafened adults to partially regain their hearing ability [1]. However, clinical outcome, most importantly speech perception, varies greatly across users. Patterns of cortical plasticity caused by deafness on one hand and partially restored input on the other may help to explain the large degree of variability. It is known that extended periods of sensory deprivation induce cortical plasticity. In particular, the lack of auditory input has been shown to induce reorganization of the auditory cortex for visual processing not only in deaf-born individuals [2] but also in postlingually deafened individuals [3]. On the other hand it has been shown that successful speech perception depends on the adaptive plasticity to the new electrical input from the CI [4]. Accordingly, it is of clinical relevance to understand whether postimplantation adaptation in postlingually deafened adults completely reverses the preimplantation reorganization of auditory cortex and, if not, whether residual preimplantation reorganization of auditory cortex is beneficial or detrimental for speech perception with a CI.

There is evidence for a visual takeover type of reorganization in the auditory cortex of CI users. Specifically, visual-evoked activation in the auditory cortex of CI users has been observed to be larger than normal hearing (NH) controls. Furthermore, the visual-evoked activation in the auditory cortex of CI users has been shown to be modulated by luminance ratio and has been inversely related to speech recognition ability with the CI [5]. This suggests firstly that the reorganization of auditory cortex that took place prior to CI implantation may be only partially reversed after CI implantation. Secondly, the study implies that elevated visual-evoked activation in the auditory cortex may impede the optimal adaptation to the CI input after implantation.

Functional reorganization in CI users seems not to be restricted to the auditory cortex but has also been observed in the visual cortex. A positron emission tomography (PET) study [6] has revealed that when presented with auditory stimuli alone, CI users showed higher activation in the visual cortex when compared with NH participants. Furthermore, the auditory-evoked activation in the visual cortex increased over time after implantation and became stimulus-specific towards potentially meaningful sounds, in particular words, syllables, and environmental sounds. The increase in auditory-evoked activation in the visual cortex was associated with CI usage duration as well as the increase in CI speech performance. These results suggest that a reorganization of the visual cortex may help to compensate for the coarse auditory input provided by the implant. Accordingly, this pattern of reorganization is potentially beneficial for CI speech performance outcome.

To summarize, previous literature suggests reorganization of auditory cortex for visual processing and reorganization of visual cortex for auditory processing in CI users. The two types of reorganization appear to have opposing effects on CI speech performance: reorganization of the auditory cortex is associated with a decrease of speech performance [5], while reorganization of the visual cortex is associated with an increase of speech performance [6]. However, no study so far has simultaneously measured both types of reorganization. Therefore it remains unclear if the maladaptive reorganization of the auditory cortex can be compensated by the beneficial reorganization of the visual cortex. In this study we investigated how the combination of reorganization of visual and auditory cortex within the same CI user jointly affects the CI speech performance. Specifically, based on previous literature we hypothesized that CI users with a higher level of reorganization of the visual cortex compared to reorganization of the auditory cortex would perform better than CI users with the opposite pattern.

In order to assess visual and auditory cortex activation patterns in CI users, we used functional near-infrared spectroscopy (fNIRS). fNIRS uses the absorption properties of near-infrared light in tissues to measure oxygenated (HbO) and deoxygenated hemoglobin (HbR) concentrations [7–9]. fNIRS is noninvasive and, in contrast to functional magnetic resonance imaging (fMRI) and electroencephalography (EEG), compatible with the CI device with no potential safety issue or noise contamination. Additionally, several studies with fNIRS have demonstrated promising results on visual and auditory processing in NH listeners and in CI users [10, 11]. fNIRS has also been used to investigate cross-modal reorganization in deaf individuals [12]. In a recent study with NH individuals we verified that fNIRS is suitable for the examination of cross-modal reorganization patterns [13].

**Figure 1:**
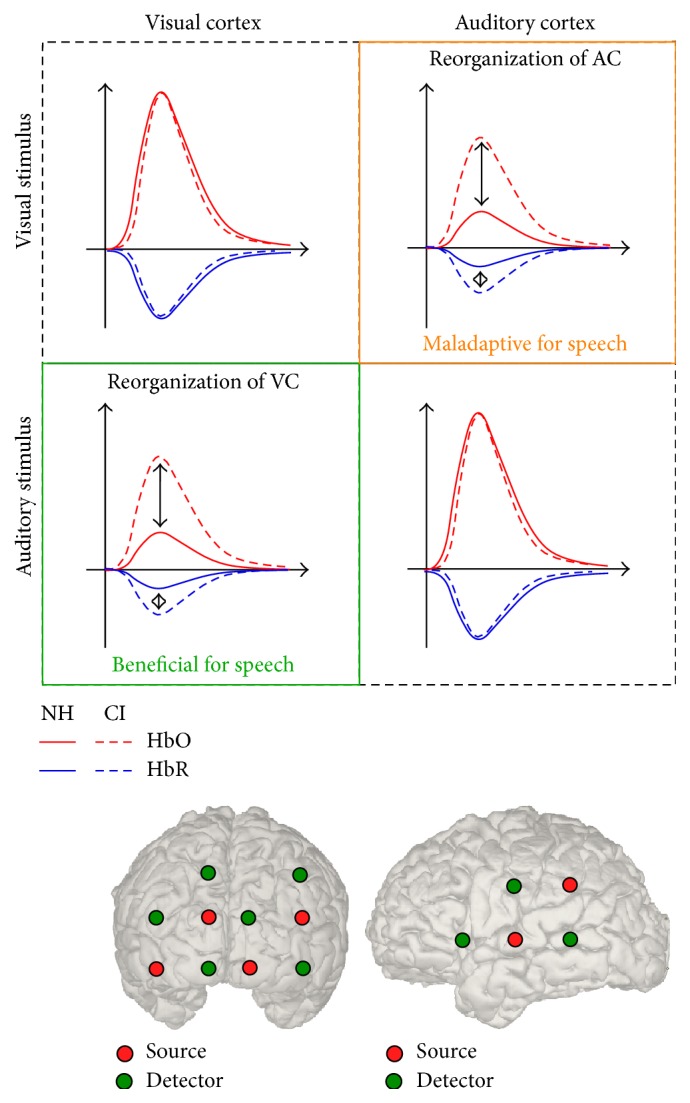
Hypothesis. The upper row represents visual stimuli and the middle row represents auditory stimuli. The left column represents measurement over visual cortex and the right column represents measurement over auditory cortex. The solid lines represent NH controls and the dashed lines represent CI users. We hypothesized observations on reorganization of both visual and auditory cortex. Reorganization of auditory cortex (upper right) is defined as the higher amount of visual-evoked activation in auditory cortex observed in CI users compared to NH controls, which has been shown to be maladaptive in terms of speech performance for CI users. Reorganization of visual cortex (lower left) is defined as the higher amount of auditory-evoked activation in visual cortex observed in CI users compared to NH controls, which has been shown to be beneficial for CI speech performance. The bottom row shows the source and detector positions mapping to the cortex using a 3D digitizer on single participant.

In the present study, we collected fNIRS data from postlingually deafened CI users and age-matched NH controls. The participants performed a visual task and an auditory task. We analyzed both the intramodal responses (visual cortex activity to visual stimuli and auditory cortex activity to auditory stimuli) and the cross-modal responses (auditory cortex activity to visual stimuli and visual cortex activity to auditory stimuli). Firstly, we hypothesized increased cross-modal responses of CI users compared to NH (Figure 1). Secondly, we studied the relationship between the level of speech perception and the degree of the joined cross-modal activation of visual and auditory cortex.

## 2. Materials and Methods

### 2.1. Participants

Forty adults (14 males and 26 females) participated in the study. Four participants were left-handed and the others were right-handed according to the Edinburgh Handedness Inventory [14]. All participants had normal or corrected-to-normal vision, and none had a history of neurological or psychiatric illness. Twenty of the participants were postlingually deafened CI users. One CI user rested his head toward the back of the chair and had to be excluded due to the resulting signal distortions. The remaining 19 CI users had unilateral implants, with three of them implanted in the left ear and the others implanted in the right ear (Table 1). All CI users had been continuously using their devices for at least 6 months prior to the experiment (mean 5.03 ± 3.75, range 0.5 to 16 years). Because of the considerable age variance across the CI users (mean 54.58 ± 14.96, range 24 to 77 years), each CI user was matched with a NH participant for gender, age, and handedness. The NH participants (mean 54.89 ± 15.80, range 24 to 78 years) served as controls and were tested for hearing ability. One NH participant was excluded due to extensive movement during experiment. All participants gave written consent prior to the experiment. All procedures were approved by the local ethics committee and conformed to the declaration of Helsinki. The participants were paid for their participation.

### 2.2. Stimuli and Setup

**Figure 2:**
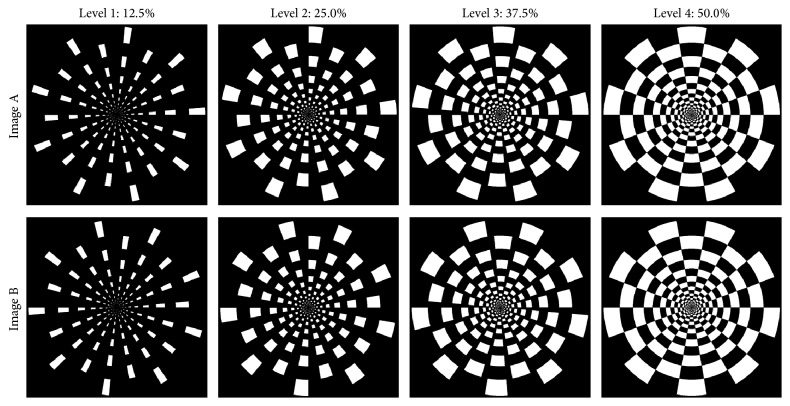
Flicking checkerboard pairs. The proportion of white pixels in the stimulus was 12.5, 25, 37.5, and 50% of the circular panel (from left to right). Image B is generated by rotating Image A by 180.

The experiment included a visual and an auditory session. For the visual session we adopted the stimuli from a previous study [5]. The visual stimuli consisted of reversing displays of circular checkerboard patterns (Figure 2). The image pair of each stimulus is referred to as Images A and B. Image B was generated by rotating Image A by 180 degrees. All stimuli (1280 × 1024 pixels) were radial in nature and consisted of 20 rings, each of which was divided into 18 sectors with neighboring sectors being of opposite color. The radial nature of the stimuli compensated for the increase in receptive-field size with eccentricity [15, 16]. There were four different pairs of checkerboard patterns that systematically varied in terms of luminance ratio: Level 1 corresponds to 12.5% white pixels, Level 2 corresponds to 25% white pixels, Level 3 corresponds to 37.5% white pixels, and Level 4 corresponds to 50% white pixels. The contrast between white and black pixels was identical in all images used. Images A and B were presented at a reversal rate of 2 Hz for 10 seconds. All visual stimuli were presented on a 24-inch monitor at a distance of 150 cm. The visual angle of the checkerboard diameter was 10.5°.

For the auditory session, we used three types of sound. The first type was four German words (Bildung, Hoffnung, Marke, and Vorteil) adopted from a previous study [17]. All words were disyllabic and matched in intensity (amplitude normalization); they were sampled at 44.1 kHz and adjusted to a duration of 800 ms. The second type was the same words reversed. The reversed words had the same long-term spectral properties but lacked intelligibility. Each word/reversed word train consisted of 3 consecutive words/reversed words and the interstimulus interval was 1.3 s. Within each word/reversed word train, the words/reversed words were either all identical or all different. The third type of sound was tone bursts. Since the current study focused on the speech stimuli, the details and the results for tone bursts were included in Supplementary Material available online at http://dx.doi.org/10.1155/2016/4382656. Thus for the auditory task we had in total 4 conditions: repeated words, unrepeated words, repeated reversed words, and unrepeated reversed words. The four conditions were implemented for the investigation of auditory adaptation and will be discussed elsewhere. Here we focused on testing what type of auditory stimuli would show higher cross-modal responses in CI users compared to NH controls. Therefore the repeated and unrepeated conditions were averaged for words and reversed words. All auditory stimuli were delivered to the participants through free-field speakers located bilaterally in front of the participants and were adjusted individually to their comfortable loudness level.

### 2.3. Experimental Design

For the visual task, 40 trials were presented (4 luminance ratios × 10 repetitions). Each trial consisted of one luminance ratio (i.e., one image pair of the reversing checkerboard pattern) presented for 10 seconds, followed by a 20-second baseline with a fixation cross in the middle of the screen. Participants were instructed to fixate on the middle of the screen during the stimuli and the baseline period. The visual task lasted for 20 minutes, and a break of 1 minute was given after 10 minutes. For the auditory task, 1 (5 consecutive 1 kHz tones) × 30 (repetitions) + 2 (3 consecutive real/reversed words) × 2 (repeated/unrepeated) × 15 (repetitions) = 90 trials were presented. The word-related trials consisted of a three-word sequence presented for 5 seconds followed by 15 seconds of silence. A silent documentary (showing animals in the wild) was presented in the middle of the screen throughout the entire session, and participants were instructed to fixate at the middle of the screen and to avoid saccades as much as possible. The auditory task took 30 minutes. All stimuli were presented in randomized order, and the order of the visual and the auditory task was counterbalanced across participants.

### 2.4. Procedure

Before the start of the experiment, all participants passed a Landolt C vision test with visual acuity more than 0.6. All NH controls passed a hearing threshold test with less than 30 dB hearing loss in each ear (125–4000 Hz). All participants were asked to answer a set of questionnaires including handedness and health state. CI participants additionally answered a questionnaire consisting of CI-related questions such as the duration of deafness and duration of CI usage. After the questionnaires, participants received an instruction sheet for both the visual and the auditory task. In the visual task, they were required always to fixate on the middle of the screen and to press a button at the end of the stimulus to indicate whether the stimulus belonged to a higher (level 3 or 4) or to a lower (level 1 or 2) luminance ratio. Prior to the actual data recording, the participants received training until a hit rate of at least 75% was reached.

In the auditory task, participants were instructed to fixate at the center of the screen and to avoid saccades as much as possible (closing the eyes was not allowed). The task was to focus on the documentary and to ignore the sound. In order to make sure that participants attended to the video and not the auditory stream, participants were told that after the experiment a questionnaire would be given related to the documentary and the answers would be evaluated. After the experiment, participants performed a questionnaire about the content of the film. Additionally, both NH controls and CI participants performed the Oldenburg sentences test [18] to evaluate their speech performance. The Oldenburg sentences test (OLSA) measures speech recognition ability with a sentence format in both silent and noise environments. The OLSA test in quiet environment (OLSA_q) measures the percentage of correct answers within a sentence at the sound intensity level of 65 SPL. The OLSA test in noise environment (OLSA_n) uses an adaptive procedure to estimate the signal-to-noise ratio at which the participants achieve 50% correct rate of sentence recognition. For CI users who failed to reach 50% correct rate in the OLSA_q test, the OLSA_n was not measured to avoid potential frustrations of the participants. As a result, one of the CI users was not tested with the OLSA_n test (Table 1).

### 2.5. Data Recording

Functional near-infrared spectroscopy (fNIRS) was recorded by a NIRScout 816 device (NIRx Medizintechnik GmbH, Berlin, Germany) with 8 LED sources (intensity 5 mW/wavelength) and 12 detectors placed on the temporal and occipital areas of the scalp (Figure 1). Regions of interest were defined as the left and the right visual area (occipital area) and as the left and the right auditory area (temporal area). Above each area, two sources and three detectors were placed. The distance between a source and its neighboring detector was 3 cm. Each source-detector pair at 3 cm distance formed a channel, resulting in five channels per area and 20 channels in total. The emitted light from sources had wavelengths of 760 nm and 850 nm, and the sampling rate was 6.25 Hz. Electroencephalography was recorded simultaneously with fNIRS and will be reported elsewhere.

### 2.6. Data Processing

The fNIRS data were imported into Matlab and were transformed to concentration levels (unit: mM) of HbO and HbR using the NILAB toolbox (NIRx Medizintechnik GmbH, Berlin, Germany). HbO and HbR concentrations were then high-pass filtered at 0.015 Hz. For the visual task, the concentrations were low-pass filtered at 0.08 Hz and for the auditory task, the concentrations were low-pass filtered at 0.1 Hz. Discrepancies in the filtering are due to the different task frequency, which is 0.03 Hz for the visual task and 0.05 Hz for the auditory task. Motion artifact was eliminated by excluding trials with concentration changes of more than 4 standard deviations away from the mean. HbO and HbR concentrations were modeled separately with the general linear model (GLM) using a Boynton canonical hemodynamic response function with 6-second delay [19, 20]. The beta values from the contrast of all stimuli (i.e., all conditions) versus baseline (corresponding to the fixation cross between the trials) were then extracted from the model. In accordance with our previous study [13], we selected within each predefined area (left/right visual area and left/right auditory area, 5 channels per area) the channel with the highest beta value independently for HbO and HbR (4 areas × 2 measurements (HbO and HbR), i.e., 8 channels were selected for each subject). Data from the selected channels were epoched from −5 s to 25 s around the onset of the stimuli for the visual task and from −2 s to 20 s for auditory task due to the different duration of visual and auditory stimuli (10 s for visual and 5 s for auditory). All trials were averaged across each condition separately. Baseline correction was applied from −5 s to 0 s for the visual task and from −2 s to 0 s for the auditory task. Grand averages across subjects were calculated for the left and the right hemisphere, for both auditory and visual areas. HbO and HbR concentrations were calculated as the mean amplitude within a time window with the length identical to the stimuli (10 s for visual task and 5 s for auditory task). The time window was defined separately for each condition by the peak latency (defined from the grand average) plus and minus half of the stimuli length (5 s for visual task and 2.5 s for word-related sounds). Since previous studies have indicated a double peak activation pattern for repeated auditory stimuli [13, 21], for responses that showed double peak pattern in the current experiment, only the first peak associated with the onset of the stimuli was used in the calculation of HbO and HbR concentrations. These concentration values were later subjected to the analysis of variance (ANOVA).

In order to assess the amount of reorganization of visual and auditory cortex, a data-driven approach was used. Specifically, since it is not well understood whether the cross-modal responses higher in CI users than NH controls show any lateralization [22], we assumed that the hemisphere showing the stronger difference between CI users and NH controls best represents the reorganization. Additionally, we defined the amount of reorganization as the relative activation between the cross-modal activation and the intramodal activation; that is, the reorganization of auditory cortex was calculated as the visual-evoked activation in auditory cortex divided by the visual-evoked activation in the visual cortex (ReorgAC = *V*_stim_ *A*_area_/*V*_stim_ *V*_area_) and the amount of reorganization of visual cortex was similarly calculated as the auditory-evoked activation in visual cortex divided by the auditory-evoked activation in the auditory cortex (ReorgVC = *A*_stim_ *V*_area_/*A*_stim_ *A*_area_). This procedure also served the purpose of normalization to account for individual differences.

### 2.7. Statistical Analysis

All statistical analyses were performed separately for HbO and HbR. To investigate the group difference between CI and NH in the visual-evoked activation in auditory cortex, a mixed factorial three-way ANOVA was performed, with condition (4 luminance ratios) and hemisphere (left, right) as within-subject factors and group (CI, NH) as between-subjects factor. Similarly for the investigation of group difference on the auditory-evoked activation in visual cortex, a mixed factorial three-way ANOVA was performed, with intelligibility (words and reversed words averaged over repeated and unrepeated conditions) and hemisphere (left, right) as within-subjects factors, and group (CI, NH) as between-subjects factor. Significant main effects and interactions were again followed up with post hoc *t*-tests, and the Huynh-Feldt correction was applied in cases of violation of the sphericity assumption.

To investigate the joint influence of reorganization of visual and auditory cortex on CI speech performance, the ReorgVC was subtracted from ReorgAC. The difference was then correlated with speech performance with Pearson's *r* correlation. Since most of our CI users performed above 75% with only one exception performing at 21.3% (Table 1) on the OLSA_q test, the OLSA_q test score was not well distributed for the purpose of correlation. Therefore we used only the results of OLSA_n test scores for the correlation. For NH controls, ReorgAC and ReorgVC were correlated separately with OLSA_n test scores with Pearson's correlation to investigate potential cortical changes related to speech-in-noise hearing ability.

## 3. Results

### 3.1. Movie Questionnaire

The questionnaires for the documentary video from all participants were evaluated by computing the rate of correct answers. Mean performance was 87.23%  ±  0.09 for CI users and 89.08%  ±  0.05 for the NH participants, indicating a high level of attention to the documentary during the auditory task. There was no significant difference between the groups (*p* > 0.4).

### 3.2. Cross-Modal Responses in CI Users and NH Controls

**Figure 3:**
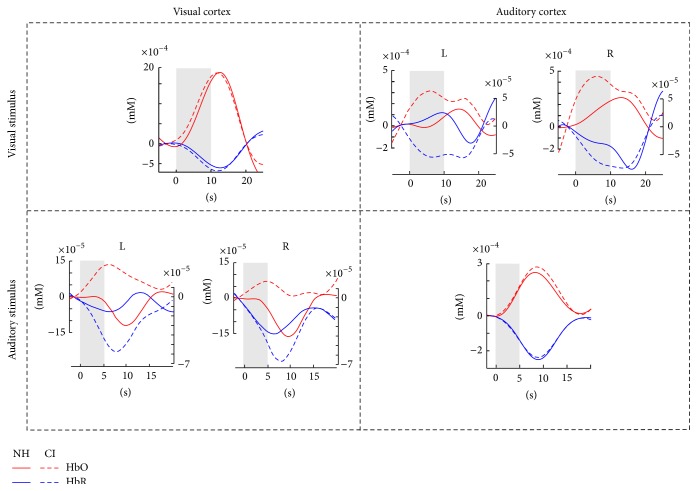
Grand averages of HbO and HbR concentrations for visual and auditory stimuli. The overall layout is identical to Figure 1. The upper row represents visual stimuli and the lower row represents auditory stimuli, both averaged over all conditions. The left column represents measurement over visual cortex and the right column represents measurement over auditory cortex. For the cross-modal responses (upper right and lower left), the activations are plotted separately for left and right hemispheres. Additionally, HbO and HbR concentrations are plotted with separate scales, the right for HbR and the left for HbO. For the intramodal responses (upper left and lower right), the activations were averaged across left and right hemispheres. One can see from the plot that the intramodal responses show almost no difference between groups, whereas the cross-modal responses show larger activation for CI users compared to NH controls.

Figure 3 shows the grand averages of HbO and HbR concentration changes separately for CI and NH groups. As previously validated [13], visual stimuli activated the visual area more than the auditory area, and auditory stimuli activated the auditory area more than the visual area for both CI and NH groups (see Supplementary Material). Consistent with our hypothesis, CI users showed higher visual-evoked activation in the auditory area and higher auditory-evoked activation in the visual area compared to NH participants. Higher visual-evoked activation in the auditory area was particularly prominent with the first peak within the stimulus time window (0 to 10 s after onset of the stimulus). These observations were confirmed by the statistics. For the visual-evoked activation in auditory cortex, a three-way ANOVA (condition, hemisphere, and group) with HbO as dependent variable showed significant main effects of the factors hemisphere (*F*(1,36) = 11.533, *p* = 0.002, and *ŋ* ^2^ = 0.911) and condition (*F*(2.45,88.34) = 3.45, *p* = 0.027, and *ŋ* ^2^ = 0.70) and a group main effect (*F*(1,36) = 3.82, *p* = 0.059, and *ŋ* ^2^ = 0.48). Two-way follow-up ANOVAs (hemisphere, condition) were performed separately for the CI users and the NH controls to follow up the group main effect. These analyses revealed a significant main effect of hemisphere (*F*(1,18) = 4.81, *p* = 0.042, and *ŋ* ^2^ = 0.55) and condition (*F*(1,62,29.19) = 3.57, *p* = 0.050, and *ŋ* ^2^ = 0.56) in CI participants. For NH listeners, only a significant main effect of hemisphere (*F*(1,18) = 7.78, *p* = 0.012, and *ŋ* ^2^ = 0.75) was found. Accordingly, a significant modulation by visual luminance ratio was found in the auditory area of CI users only. To investigate whether the hemisphere main effect contributed to the group difference, independent follow-up *t*-tests were performed separately for left and right auditory areas which compared concentration levels between the two groups. The results indicated differences in both the left auditory area (*t*(36) = 1.98, *p* = 0.056) and the right auditory area (*t*(36) = 1.82, *p* = 0.078), suggesting a group difference regardless of hemisphere and condition. Therefore, HbO concentrations were averaged over all conditions and hemispheres for evaluating the correlation with speech performance.

For the visual-evoked activation in auditory cortex with HbR, the three-way ANOVA revealed a significant group effect (*F*(1,36) = 7.21, *p* = 0.011, and *ŋ* ^2^ = 0.74). The significant group main effect confirmed the functional reorganization of auditory cortex in CI users. Two-way ANOVA was performed separately for CI and NH groups to follow up the group main effect. The results revealed a significant main effect of hemisphere (*F*(1,18) = 7.78, *p* = 0.001, and *ŋ* ^2^ = 0.75) in NH participants but not in CI users, where NH controls showed enhanced activation in the right auditory area compared to the left. This suggested that the group main effect was mostly driven by the left auditory area. To verify this, follow-up independent-samples *t*-tests were performed to test the group difference separately for left and right auditory areas. The results showed a significant group difference only in the left auditory area (*t*(36) = −2.79, *p* = 0.008) and not in the right (*t*(36) = −1.64, *p* = 0.110), confirming that the group difference was due to the reduced activation in the left auditory area in NH controls. Given the lack of a condition main effect and the significant group difference for the left hemisphere, we computed the average of the HbR concentrations over all conditions but performed the correlation analysis only for the data from the left auditory area.

For the auditory-evoked activation in visual cortex, no significant effect was found for the three-way ANOVA (intelligibility, hemisphere, and group) with HbO. On the other hand, the three-way ANOVA with the dependent variable HbR concentration revealed a significant main effect of group (*F*(1,36) = 5.17, *p* = 0.029, and *ŋ* ^2^ = 0.60). The results confirmed reorganization of visual cortex for words as well as for reversed words. Two-way ANOVAs were performed separately for CI and NH participants to follow up the group effect. The results showed no significant effect of hemisphere for the CI users but a significant effect of hemisphere for the NH participants (*F*(1,18) = 6.23, *p* = 0.022, and *ŋ* ^2^ = 0.66), which was due to reduced responses in the left compared to the right hemisphere. This suggests that the group main effect was mainly the result of the difference in the response from the left visual area. To verify this, follow-up independent-samples *t*-tests were performed to test the group difference separately for the left and the right visual areas. The results showed a significant group difference only in the left visual area (*t*(36) = −3.15, *p* = 0.003) and not in the right visual area (*p* > 0.1), confirming that the group difference was mainly driven by response differences in the left visual area. Therefore, for the correlation with the speech performance, we averaged HbR concentrations over all conditions measured in the left visual area.

### 3.3. Correlations with Speech Performance

The results for the amount of reorganization of auditory cortex (ReorgAC = *V*_stim_ *A*_area_/*V*_stim_ *V*_area_) and the reorganization of visual cortex (ReorgVC = *A*_stim_ *V*_area_/*A*_stim_ *A*_area_) are listed in Table 2. The ReorgAC was computed for both HbO and HbR concentrations due to the higher visual-evoked activation in auditory cortex for CI users compared to NH controls. On the other hand, the ReorgVC was calculated only for the HbR due to the lack of significant results with HbO concentration.

**Figure 4:**
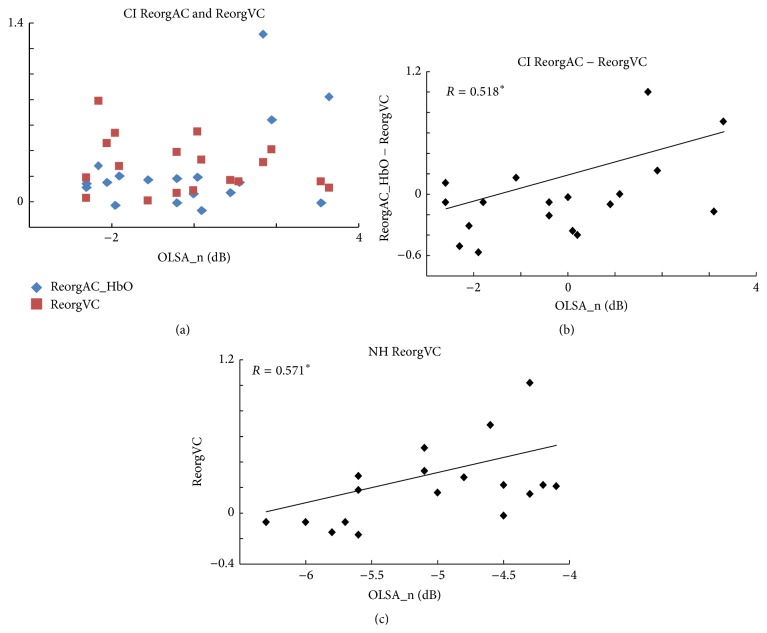
Correlations between the cross-modal reorganizations and the speech performance. (a) ReorgAC_HbO and ReorgVC plotted separately for each CI user according to their speech performance. (b) Correlation between the difference of ReorgAC_HbO and the ReorgVC and the test score for OLSA speech in noise (OLSA_n). Note that the lower signal to noise ratio threshold for OLSA_n test represents a better performance. (c) Correlation performed with NH controls. The *y*-axis is the ReorgVC and the *x*-axis is the OLSA_n test score.

Figure 4(a) shows the ReorgAC and ReorgVC separately for individual CI users based on their speech performance. To explore the joint influence of ReorgAC and ReorgVC on CI performance, the ReorgVC was subtracted from the ReorgAC_HbO and ReorgAC_HbR separately. The correlation with the OLSA_n test scores was significant (*R* = 0.518, *p* = 0.027, Figure 4(b)) for HbO but not for HbR (*p* > 0.5). This result suggests that CI users with higher beneficial reorganization of visual cortex than maladaptive reorganization of auditory cortex perform better than CI users with the opposite pattern. For NH controls, neither the ReorgAC_HbO nor ReorgAC_HbR were correlated with the OLSA_n test scores (*p* > 0.2). On the other hand, a significant correlation was found between the ReorgVC and the OLSA_n test scores (*R* = 0.571, *p* = 0.011, Figure 4(c)), indicating that, during the processing of auditory stimuli, a higher activation in visual cortex is related to poorer speech-in-noise performance.

## 4. Discussion

The current fNIRS study confirmed former observations of higher visual-evoked activation in auditory cortex and higher auditory-evoked activation in visual cortex in CI users compared to NH controls [5, 6]. The results demonstrated functional reorganization of auditory cortex for visual processing and of visual cortex for auditory processing in CI users. Importantly, we showed first evidence of the joint influence of both types of reorganization on CI speech performance. Specifically we found that CI users with more reorganization of visual cortex compared to reorganization of auditory cortex perform better than CI users with the opposite pattern. Our results also suggest that, in the group of NH controls, the higher auditory-evoked activation in visual cortex is related to worse speech in noise performance, suggesting an early onset of cortical changes for minor hearing loss in speech-in-noise ability.

### 4.1. Cross-Modal Responses in CI Users and in NH Controls

The observation of significantly higher visual-evoked activation in auditory cortex in CI users compared to NH controls confirmed former observations of reorganization of auditory cortex in CI users [5]. This suggests that* postimplantation* adaptation to the implant signal did not completely reverse the reorganization of auditory cortex that was caused by sensory deprivation prior to the CI implantation. As the visual-evoked activation in the auditory cortex of CI users was modulated by the luminance ratio of the visual stimuli, one can assume that the auditory cortex might still serve some functional purpose for visual processing.

Similarly, our results also revealed higher auditory-evoked activation in visual cortex in CI users compared to NH controls, suggesting reorganization of visual cortex of CI users, as observed in previous studies [6, 23]. Whereas previous studies showed reorganization of visual cortex to sounds including syllables, words, and environmental sounds, our results extend those findings to pure tones (see Supplementary Material) and reversed words. This suggests that intelligibility is not a requirement for the elicitation of visual recruitment for auditory stimuli in CI users. Additionally, since the current experiment implemented a passive auditory task, our results also suggested that attention is not necessary for the elicitation of visual recruitment during auditory processing. This was further verified by the results on the questionnaire showing a high level of concentration on the video. One may argue that the presence of the visual stimuli might cause a potential confounder in the current experiment. However, the same was applied to the NH controls and therefore the observed difference between CI and NH groups could not be the result of such a confounder.

The observed cross-modal activation cannot be attributed to tinnitus, since no difference was found between the CI users without tinnitus and the CI users perceiving tinnitus (see Supplementary Material). Additionally, lateralization effects were observed in the current study. Specifically, with HbR concentration changes, larger group differences in visual-evoked activation in auditory cortex and, conversely, larger differences in auditory-evoked activation in the visual cortex were observed mostly in the left hemisphere. Several studies investigating cross-modal reorganization have observed higher visual-evoked activation in auditory cortex in prelingually deafened individuals compared to NH controls mostly in the right hemisphere [24–26]. Our observation of group difference over the left hemisphere might thus be considered surprising. However, the current study investigated postlingually deafened CI users, and it is likely that lateralization in plastic functional changes in the auditory cortex differs between pre- and postlingually deafened individuals. A recent study has shown that the reorganization of the left auditory cortex is more associated with sign language rather than auditory deprivation in general, which is consistent with the idea that language experience could contribute to the lateralization effect [27]. Although a few studies have also investigated cross-modal reorganization in postlingually deafened CI users, discrepancies such as type of visual stimuli [28, 29] and side of implantation of CI users [5, 29] make it difficult to directly compare the results on lateralization. It is likely that the lateralization of cross-modal reorganization is stimuli or implant side dependent. Note also in the current study that the lack of group difference in the right hemisphere is mostly driven by the NH controls showing larger visual-evoked activation in the auditory cortex and auditory-evoked activation in the visual cortex in the right compared to the left hemisphere. The observed pattern in the current study was not observed with our previous study with younger NH participants [13], and therefore it is also possible that an age effect contributes to the observed lateralization of plastic changes in visual and auditory cortex. Since lateralization for cross-modal reorganization is not well understood, more studies are required to systematically examine the hemispheric asymmetry of cross-modal activation in the auditory and visual cortex of early and late deafened CI users.

We observed for the NH controls a relation between auditory-evoked activation in visual cortex and speech-in-noise performance. This is consistent with a recent finding suggesting early onset of cortical reorganization with hearing loss [30, 31]. Importantly, minor hearing problems as detected by speech-in-noise tests could already induce changes of cortical functionality. Further studies are required to identify the relationship between cross-modal cortical reorganization, mild hearing loss, and speech-in-noise ability.

### 4.2. Influence of Reorganization of Visual and Auditory Cortex for CI Outcome

**Figure 5:**
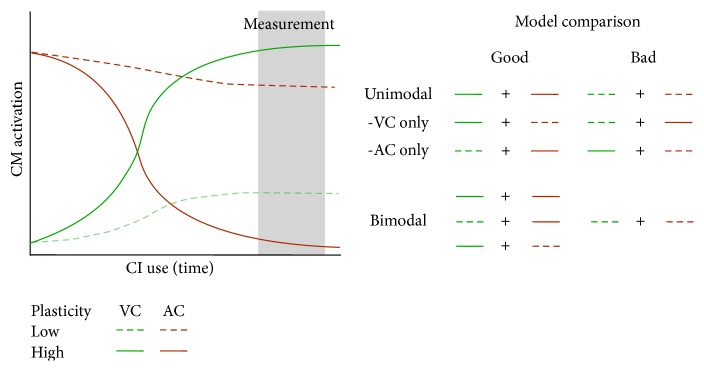
Model of the influence of cross-modal activation of auditory and visual cortex on CI performance. The *y*-axis shows the amount of cross-modal activation and the *x*-axis shows time after CI implantation. The brown lines represent cross-modal activation in auditory cortex (AC), which is assumed to be maximal at time of implantation and reduces with time. The green lines represent cross-modal activation of visual cortex (VC), which develops rapidly after implantation, when auditory input is restored. The dashed lines indicate low plasticity as expressed with small changes after implantation. The solid lines indicate high plasticity as expressed with large changes after implantation. The area between the respective solid and dashed lines represents the potential variation in plasticity. When considering plasticity levels in visual and auditory cortex* together* (Bimodal), CI users with a solid green line and a dashed brown line or with a solid brown line and a dashed green line would both be considered good performers since in both cases cross-modal activation in visual cortex is higher than in auditory cortex. However, if one considers each sensory modality* separately* (Unimodal), the prediction would vary depending on the sensory modality considered. Specifically, when considering visual cortex only, one would expect the CI user with a solid green line and a dashed brown line to perform significantly better than the CI user with a solid brown line and a dashed green line. However when considering auditory cortex alone, one would expect the opposite. This ambiguity is resolved with the currently proposed Bimodal model.

The current data show that a beneficial reorganization of visual cortex could compensate for the maladaptive reorganization of auditory cortex, to reach good performance in speech recognition in CI users. Firstly, the results suggest that CI users show different amounts of plasticity in auditory and visual cortex. Since the reorganization of auditory cortex decreases over time and reorganization of visual cortex, in contrast, increases over time after CI implantation, it can be concluded that the amount of change is not identical if, for the same CI user, a similar high or low level of reorganization of visual and auditory cortex is observed (Figure 4(a)). Secondly, our results also suggest that as long as at least one sensory modality shows a great amount of plasticity, individuals may perform well with the CI. By considering plasticity in both sensory modalities together, the ambiguity of performance of CI users whose reorganization is similarly high or similarly low in visual and in auditory cortex would be resolved. See Figure 5 for a schematic illustration. On the other hand, one should note here that the levels of reorganization of auditory and visual cortex were both found significantly higher in CI users compared to NH controls. As a result, the amount of cross-modal activation did not return to normal levels, even in good CI performers. Instead, the level of both maladaptive reorganization of auditory cortex and beneficial reorganization of visual cortex remained higher for good CI performers.

Previous literature has reported several potential causes for the large variations of CI clinical outcome in speech performance. In particular, findings have suggested that this variability is related to the implant device and electrical stimulation of the auditory nerve, the amount of surviving spiral ganglion cells, and the degree of cortical plasticity in the central auditory system [32–34]. In the period of deafness, the auditory nerves deteriorate [33] and cross-modal reorganization takes place [2]. Thus, the preimplantation deafness plays an important role in the clinical outcome. Specifically, the longer the duration of deafness is, the worse the outcome would be for speech performance with CI [1, 35]. Additionally, the onset age of deafness is also found relevant for the clinical outcome, as postlingually deafened participants usually benefit more than prelingually deafened participants who failed to receive a CI before the age of seven [36–38]. To summarize, most studies so far have implied that the CI outcome has been primarily determined by the factors related to the time prior to the CI implantation. On the other hand, our data has suggested that the plasticity that took place after the CI implantation is, if not more, at least not less important than the plasticity before the implantation. In particular, we have shown that the postimplantation plasticity could potentially compensate the preimplantation plasticity to reach a satisfactory speech performance with a CI. Therefore we suggest that postimplantation plasticity is an additional factor for the estimation of clinical outcome of CI [36]. Nevertheless, independent studies are required for further verification. In particular, a prospective longitudinal study including pre- and postimplantation observations, focusing on the changes of cross-modal activation in both auditory and visual cortex, would best test our prediction. Few studies have targeted the dynamics of cortical adaptation after implantation [29, 34, 39, 40], but dynamic plasticity changes of reorganization in visual and auditory cortex before and after implantation within the same CI user have not been addressed. While other neuroimaging techniques such as fMRI (safety issue), PET (radiation exposure), and MEG (artifact) are not suitable to address this question, the present study and previous work from our lab [13] highlight the value of fNIRS in this context.

### 4.3. Mechanisms of Cross-Modal Reorganization

Several potential mechanisms have been suggested to mediate cross-modal reorganization. One possibility would be the direct anatomical connection between visual and auditory cortical areas [41, 42]. Specifically, in auditory cortex it has been reported with single-unit recording that the primary auditory area contains neurons responding to nonauditory input [41]. Therefore the cross-modal reorganization observed in CI users could simply be an enhancement of preexisting connections between the sensory modalities or the increase of units related to the other sensory modality, thereby reducing the risk of auditory neural atrophy caused by sensory deprivation. This interpretation is also in line with the current finding of a recruitment of the visual cortex for auditory processing in NH controls with worse speech-in-noise performance.

### 4.4. HbO and HbR Concentrations

The current experiment successfully replicated our previous finding of area specificity [13] with both CI users and NH participants and in both HbO and HbR concentrations. Specifically, we observed that visual stimuli activated the visual area more than the auditory area and that auditory stimuli activated the auditory area more than the visual area (see Supplementary Material). However, the analysis of cross-modal responses, that is, visual-evoked activation in auditory cortex and auditory-evoked activation in visual cortex, showed rather inconsistent results when HbO and HbR concentration effects were compared, particularly when correlations with speech performance were analyzed. The inconsistency between HbO and HbR is likely due to the difference in their signal-to-noise ratios. Several studies have reported that the HbO concentration is more affected by physiological noise such as heart beat and respiration [43, 44]. Accordingly, HbO showed in general a larger variance across subjects. This may also explain the lack of a group difference between CI and NH participants for HbO concentrations. On the other hand, HbO concentration levels often show larger responses and therefore better stimulus-related modulation effects, as can be also observed in the current results. This suggests that HbO and HbR measurements might be sensitive to different aspects of the neural responses and therefore potentially explain the inconsistency of the results.

At present, it remains unclear whether the two hemoglobin forms relate equally well with neuronal activity. Specifically, a few studies have reported auditory-evoked potentials recorded by EEG correlating with only HbO concentration changes in auditory cortex [13, 45], while others have reported visual-evoked potentials recorded by EEG correlating only or at least better with HbR concentration in visual cortex [13, 46, 47]. This is consistent with our current findings, where HbO concentration in visual-evoked activation in auditory cortex and HbR concentration in auditory-evoked activation in visual cortex were combined together and revealed a correlation with CI speech performance. Therefore it is possible that the inconsistency between HbO and HbR observed in the current study reflects different degrees of sensitivity of HbO and HbR for the measurement of visual and auditory cortex. Since only a few studies have investigated the relationship between EEG and fNIRS directly, the exact relationship between HbO and HbR patterns on one hand and neuronal activation on the other is still not well understood. Additionally, fMRI evidence suggests that experimental design could well contribute to the variation of association between neural activities and hemodynamic responses [48, 49]. More studies are required to further dissociate the relationship of HbO and HbR with neuronal activity.

### 4.5. Offset Response in the Auditory Cortex

Previous fMRI studies have demonstrated a double peak pattern in the hemodynamic response for repetitive auditory stimuli in the auditory cortex [21, 50]. This double peak was shown to be related to the strong adaptation toward the repeated stimuli, thereby creating the second peak cause by the offset of the stimuli. In line with this finding, in our previous study with fNIRS we also observed the double peak pattern in the auditory cortex for the repeating auditory stimuli [13]. This double peak pattern was not present in the current study for the auditory-evoked activation in auditory cortex. This is likely due to differences in the stimulus material. In the present study each word was repeated 3 times (stimulus duration: 5 seconds) while in the former study each tone was repeated 20 times (stimulus duration: 20 seconds). It might therefore be that the auditory stimuli we used here did not show a strong enough adaptation to induce an offset response. Interestingly on the other hand, we observed a double peak pattern for visual-evoked activation in the auditory cortex. Since in the current experiment the visual stimuli lasted for 10 seconds with 20 repetitions, our results suggest that the strong adaptation characteristic of auditory cortex was retained even after reorganization for visual processing. This observation is in line with several recent studies showing the preservation of functional specialization of auditory cortex after the visual takeover reorganization [51–54]. A detailed study systematically manipulating this form of adaptation is needed to investigate how stimulus duration and repetition rate relate to offset responses. We would predict that the double peak pattern decreases with decreasing repetitions.

## 5. Conclusions

The present fNIRS study observed residual cross-modal reorganization of the auditory cortex of CI users, which was possibly induced during the period of deafness prior to implantation. Importantly, cross-modal reorganization was not limited to the auditory cortex and was also observed in the visual cortex. We suggest that cross-modal reorganization in both auditory and visual cortices may jointly influence CI performance. CI users may perform well as long as the beneficial cross-modal activation in the visual cortex is more dominant than the maladaptive cross-modal activation in the auditory cortex.

## Supplementary Material

The Supplementary Material includes results of a comparison between intra-modal responses and cross-modal responses for both CI users and NH controls. Additionally, results for a comparison between CI users and NH controls regarding cross-modal responses for auditory tone stimuli are also listed. Finally, since it is common for CI users to perceive tinnitus, the potential influence of tinnitus on cross-modal responses is also included in the Supplementary Material.

## Figures and Tables

**Table 1:** Subject demographics of cochlear implant users.

Subject	Gender	Age	Implant ear	Duration of deafness (years)	CI usage (years)	OLSA_q (%)	OLSA_n (dB)
1	M	24	R	14	7	99,30	−2,3
2	M	47	R	0.25	3	21,30	
3	F	51	R	2	7	100,00	0,9
4	F	22	R	8	2	98,00	−2,6
5	M	63	R	2	1	94,00	1,9
6	F	67	R	6	16	96,70	−1,9
7	F	49	L	25	6	96,00	−2,1
8	M	71	R	27	5	80,70	1,7
9	F	58	R	1	10	89,30	0
10	F	57	R	10	5	98,00	−0,4
11	F	59	R	4	2	91,30	0,1
12	F	77	R	13	6	100,00	−1,1
13	M	64	R	3	3	76,00	3,3
14	F	52	L	8	5	94,70	−2,6
15	F	36	R	1	9	93,30	0,2
16	M	70	R	7	2	93,30	−0,4
17	F	58	R	2	2	82,70	1,1
18	F	46	L	5	4	100,00	−1,8
19	F	66	R	0.5	0.5	78,70	3,1

**Table 2:** Reorganizations in visual and auditory cortex.

			Mean (%)	STD (%)
ReorgAC	HbO	CI	23	34
		NH	4	17
	HbR	CI	8	14
		NH	−3	32

ReorgVC	HbR	CI	27	21
		NH	9	29
