# Supplementary material for: Cross-Modal Functional Reorganization of Visual and Auditory Cortex in Adult Cochlear Implant Users Identified with fNIRS
Source: Neural Plast. 2015 Dec 27;2016:4382656. doi: 10.1155/2016/4382656 (PMC4706950; doi:10.1155/2016/4382656)
Supplement: Supplementary file 1 — The Supplementary Material includes results of a comparison between intra-modal responses and cross-modal responses for both CI users and NH controls. Additionally, results for a comparison between CI users and NH controls regarding cross-modal responses for auditory tone stimuli are also listed. Finally, since it is common for CI users to perceive tinnitus, the potential influence of tinnitus on cross-modal responses is also included in the Supplementary Material. [file 4382656.f1.pdf]

Supplementary materials

Intra-modal versus cross-modal responses

Visual stimuli

The three-way ANOVA (area, hemisphere, group) with HbO responses as dependent variable revealed a significant main effect of area ($F(1,36)=150.55$, $p<0.001$, $\eta^2=1.00$). Similarly for HbR, the three-way ANOVA (area, hemispheres, group) also showed a significant area main effect of area ($F(1,36)=158.33$, $p<0.001$, $\eta^2=1.00$). The results confirmed significantly larger activation in the visual area for visual stimuli for both groups of participants, regardless of the measured hemisphere.

Auditory word-related stimuli

The three-way ANOVA (area, hemisphere, group) with HbO concentration revealed a significant main effect only for the area factor ($F(1,36)=14.85$, $p<0.001$, ŋ2=0.96). Similarly, the three-way ANOVA (area, hemisphere, group) with HbR concentration as dependent variable also showed a significant main effect for the area factor ($F(1,36)=109.52$, $p<0.001$, ŋ2=1.00). Both results confirmed a significantly larger activation in the auditory area than in visual for auditory stimuli for both the CI users and the NH listeners, regardless of the measured hemispheres.

Auditory tones

The auditory tone stimulus was a 1 kHz tone adopted from a previous study [1]. The 1 kHz pure tone was sampled at 44.1 kHz and lasted for 40 ms (with 5 ms linear rise/fall). The inter-stimulus interval was 0.7 s and each train of tone bursts consisted of 5 consecutive tones, summing up to a period of 3 s. During the experiment, each block consisted of 3 second tone bursts followed by 15 seconds fixation cross.

Fig. S1 illustrates the grand averages of concentration changes for auditory tones. Each epoch was baseline corrected from -2 s to 0 s. Consistent with our hypothesis, HbR concentration revealed larger activation in the auditory than in the visual area for both the CI users and the NH participants. By contrast, the results for the HbO concentration were not as clear. NH participants showed consistently smaller activity in the visual area compared with the auditory area, but the CI users showed almost identical level of activation for both sensory areas. Additionally, CI users showed higher activation in the visual area compared to NH participants for both the HbO and the HbR concentrations.

For comparison between intra-modal responses and cross-modal responses, the three-way ANOVA (area, hemisphere, group) with HbO concentration suggested a tendency in main effect of the factor area ($F(1,36)=3.32$, $p=0.077$, $\eta^2=0.43$) and interaction between the factors area and group ($F(1,36)=3.78$, $p=0.060$, $\eta^2=0.47$). To follow-up on these results, the HbO of the left and the right hemisphere were first averaged, given that there was no main effect of hemisphere. Afterwards, post-hoc independent-samples t-tests were performed between groups for both the visual and the auditory responses. In addition, post-hoc paired t-

tests were performed to compare the visual and the auditory responses separately for the two groups. The results showed a significant difference between visual and auditory responses for the NH participants ($t(18)=-2.68$, $p<0.05$) but not for the CI participants ($p>0.9$), and no significant group difference for either the visual or the auditory responses. This suggests that the CI users showed a similar level of responses in auditory and visual areas. By contrast, the NH participants showed significantly larger responses in the auditory area compared to the visual area. With HbR concentration, three-way ANOVA (area, hemisphere, group) showed a significant effect on sensory area ($F(1,36)=37.73$, $p<0.001$, $\eta^2=1$) and group ($F(1,36)=9.18$, $p<0.01$, $\eta^2=0.84$). The results revealed a significantly higher activation in the auditory area compared with the visual area regardless of the groups, confirming area specificity with auditory stimuli. Additionally, the results also indicated a significantly higher activation for the CI users than for the NH participants, regardless of the sensory area and the hemisphere, potentially reflecting a higher demand of neural resources to the CI input in CI users.

Regarding the investigation of cross-modal activation, no significant effect was found for the two-way ANOVA (hemisphere, group) with HbO concentration. On the other hand, the two-way ANOVA (hemisphere, group) with HbR concentration revealed a significant effect of group ($F(1,36)=8.83$, $p<0.01$, $\eta^2=0.82$, Fig. S2), which confirmed the observation of reorganization of visual cortex for auditory pure tones in CI users.

Effect of Tinnitus

To investigate the potential influence from tinnitus on our results, we performed an additional analysis in which we subdivided the group of CI users into two groups based on whether they reported tinnitus or not. The Mann-Whitney U test was used to compare the two groups ("No Tinnitus", "Report Tinnitus") with regards to cross-modal activations (for both visual and auditory cortex, and for both HbO and HbR). The result is shown in Fig. S3. None of the comparisons was found to be significant (all $p>0.3$), indicating that the perception of tinnitus does not influence the observed cross-modal activation in visual and auditory cortex of CI users.

Figures

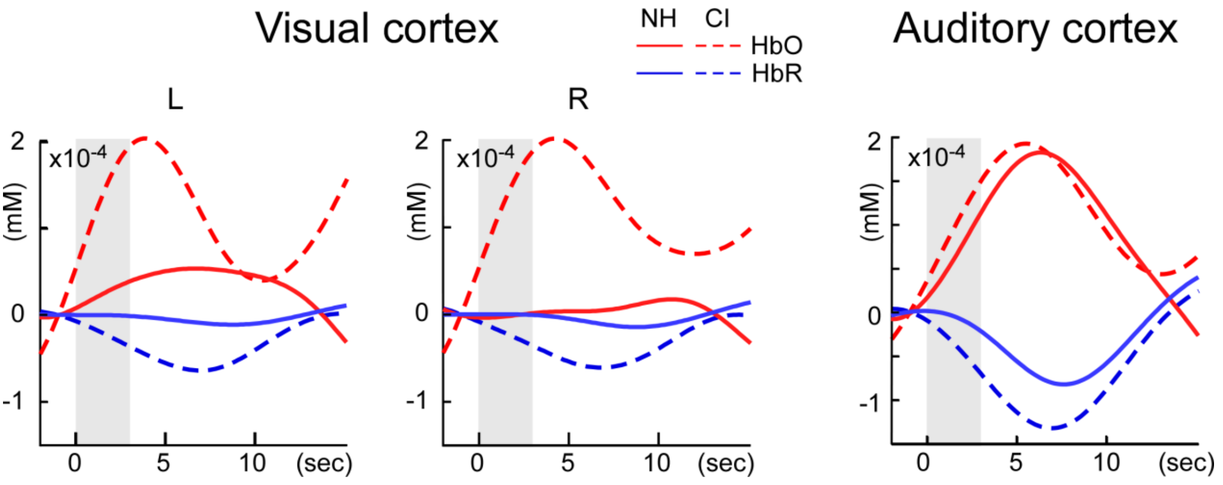

Fig. S1 Grand averages of HbO and HbR concentrations for auditory tone stimuli. The left column represents measurement separately for left and right hemisphere over visual cortex, and the right column represents measurement over auditory cortex averaged across hemispheres. One could see from the plot that the intra-modal responses show little difference between CI and NH, however, that the cross-modal responses show larger activation for CI users compared to NH controls.

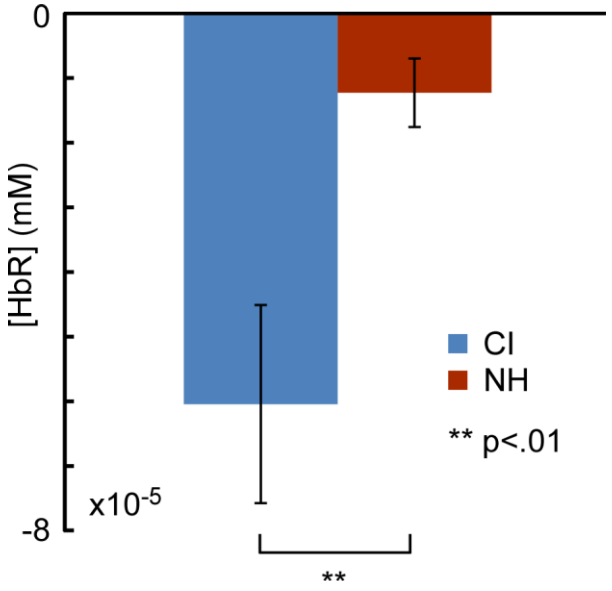

Fig. S2 HbR concentration changes for auditory-evoked activation in visual cortex. The cross-modal activation in visual cortex observed with CI users was found significantly larger than with NH controls.

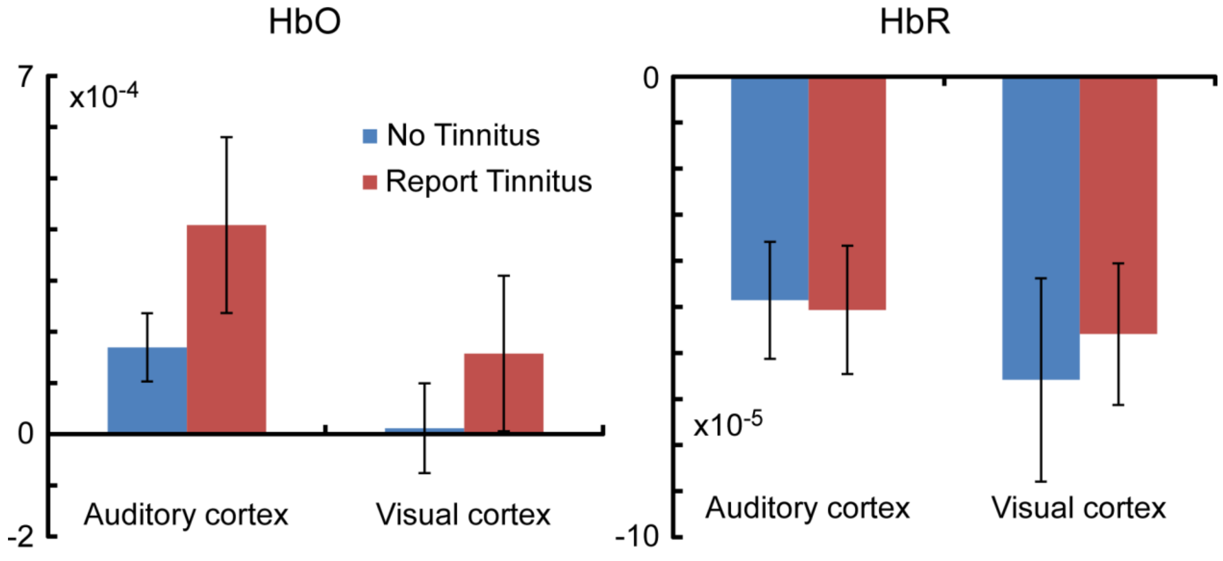

Fig. S3 Means and standard error means of HbO (left figure) and HbR (right figure) concentration changes for visual-evoked activation in auditory cortex and auditory-evoked activation in visual cortex. The values are given separately for CI users without tinnitus (blue; N=6) and CI users perceiving tinnitus (red; N=13). None of the comparisons revealed a significant group difference, suggesting that the perception of tinnitus did not affect the observed cross-modal activation in auditory and visual cortex of CI users.

Reference

1. Zhang, F., et al., *Mismatch negativity and adaptation measures of the late auditory evoked potential in cochlear implant users.* Hear Res, 2011. **275**(1-2): p. 17-29.
